# Endoscope-assisted Transoral resection of an elongated fractured styloid process in Eagle syndrome: A case report

**DOI:** 10.1016/j.ijscr.2025.111270

**Published:** 2025-04-10

**Authors:** Fares Abdullrahman, Majed Al-Ajami, Firas Abdullrahman, Jafar Hamdy, Maher Al-Ajami

**Affiliations:** a Oral and Maxillofacial Surgery Department, Faculty of Dentistry, Lattakia University, Lattakia, Syria; b Department of Otorhinolaryngology, Hama National Hospital, Hama, Syria; c Oral and Maxillofacial Surgery Department, Faculty of Dentistry, Aleppo University, Aleppo, Syria; d Department of Orthodontic, Faculty of Dentistry, Aleppo University, Aleppo, Syria

**Keywords:** Eagle's syndrome, Elongation of the stylohyoid process, Endoscope-assisted, Transoral

## Abstract

**Introduction and importance:**

Eagle's syndrome is a rare condition associated with the elongation of the styloid process or calcification of the stylohyoid ligament; it is clinically characterized by unexplained referred pain that radiates into the mandible, ear, and throat, often misdiagnosed as various cervicocraniofacial disorders.

**Case presentation:**

A 38-year-old male presented with severe, unexplained neuralgic pain that persisted for about two months. The pain worsened with patient neck movements, and some relief was achieved only through lidocaine injections in the tonsillar bed. After multiple consultations with various specialists, investigations focused on a left elongated, non-displaced fracture of the styloid process, accompanied by calcification of the stylohyoid ligament, suggesting Eagle syndrome. The patient's symptoms significantly improved after surgery, with no recurrence of pain during the subsequent follow-up period.

**Clinical discussion:**

Eagle syndrome is a rare condition that has no clear cause or specific treatment method and includes a group of nonspecific clinical symptoms.

**Conclusion:**

It is important that this condition be diagnosed well and managed professionally, as it includes and encompasses important anatomical structures.

## Highlights

• A thorough overview of Eagle Syndrome has been provided, covering diagnosis, clinical investigation, and treatment approaches.
• Treatment options for Eagle Syndrome include both medical conservative and surgical interventions.
• Due to the common, non-specific nature of the symptoms, misdiagnosis and delayed management may occur.

## 1 Introduction

Eagle or stylohyoid syndrome is named after Watt W. Eagle, an otolaryngologist, who documented the first case in 1937. This uncommon condition is associated with an elongated styloid process, which interferes with the function of adjacent structures, resulting in orofacial and cervical pain [1]. Initially, normal styloid process is approximately 2.5–3 cm long, with any process exceeding this length considered abnormally elongated. Due to its rarity and the variability in symptoms, the exact prevalence can be difficult to determine. However, it is considered approximately 4–10 % of general population [1].

The exact etiology is not clearly understood, and several theories have been proposed. These include congenital elongation, calcification of the stylohyoid ligament, the formation of bone tissue at the ligament's insertion point (sometimes in patients following tonsillectomy), local chronic irritation caused by osteitis, periostitis, or tendonitis of the styloid process and the stylohyoid ligaments, compressing cranial nerves and arteries, particularly (fifth, seventh, eighth, ninth, and tenth cranial nerves) and internal or external carotid artery, resulting in jaw, throat, neck and infra-orbital pain [2,3].

Clinically, Eagle syndrome is most commonly observed in individuals in their third and fourth decades of life. While there is no significant gender preference, symptoms tend to be more prevalent in women. The symptoms can range from mild discomfort to severe neurological referred pain [2].

The most common areas of pain were the ear (64.3 %), underneath the angle of the mandible (50 %), throat (46.4 %), and neck (30.4 %), the sensation of a foreign body in the pharynx (55 %). Over 70 % of patients reported tinnitus, dysphagia (pain while swallowing), and pain that was exacerbated by head rotation [5,7]. Additionally, a mass or bulge may be palpable in the ipsilateral tonsillar fossa, intensifying the patient's symptoms. Eagle syndrome usually manifests unilaterally, but it can occasionally present bilaterally [3,5].

The management of elongated styloid process syndrome can be either conservative (analgesics, anti-depressants, anti-convulsants, trans-pharyngeal injections of steroids and lidocaine, non-steroidal anti-inflammatory drugs, and the application of topical heat) or surgical (shortening of the styloid process) which can be performed through either an intraoral or an extraoral approach. 51 % of patients underwent surgical treatment, 92.2 % via intraoral and 7.8 % via cervical approaches [1,3,4].

This work has been reported in line with the scare criteria [7].

## 2 Case presentation

**Fig. 1:**
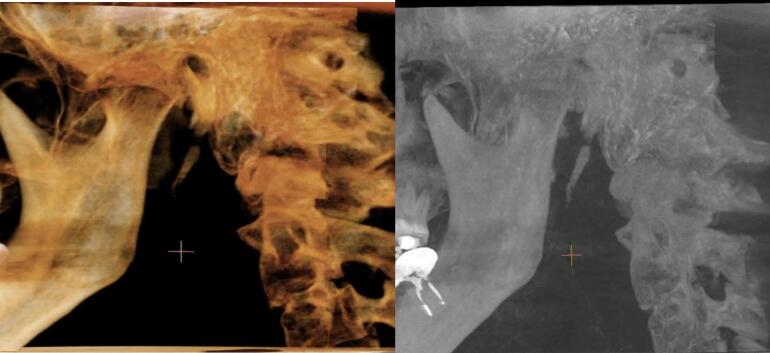
3D MDCT images of the left styloid process shows the elongation with displaced fracture.

A 38-year-old man presented with symptoms of severe left-sided unexplained neuralgic stabbing pain that radiated to the ipsilateral angle of the mandibular, throat and ear lasted about two months. The pain worsened with the patient's head movement and some relief was gained only when injecting lidocaine in the tonsillar bed. After multiple consultations with various specialists that showed no pathological changes in the oral cavity, oro-hypopharynx, larynx, soft tissues of the neck, or temporal bones, however, more detailed radiologic investigation was directed toward the left elongated non-displaced fractured styloid process with calcification of the stylohyoid ligament (Fig. 1). Multi-detector computed tomography (MDCT) with 3D reconstructions shows a 46 mm elongated fractured left styloid process compared to the contralateral side (Fig. 1), leading to the diagnosing of Eagle's syndrome.

**Fig. 2:**
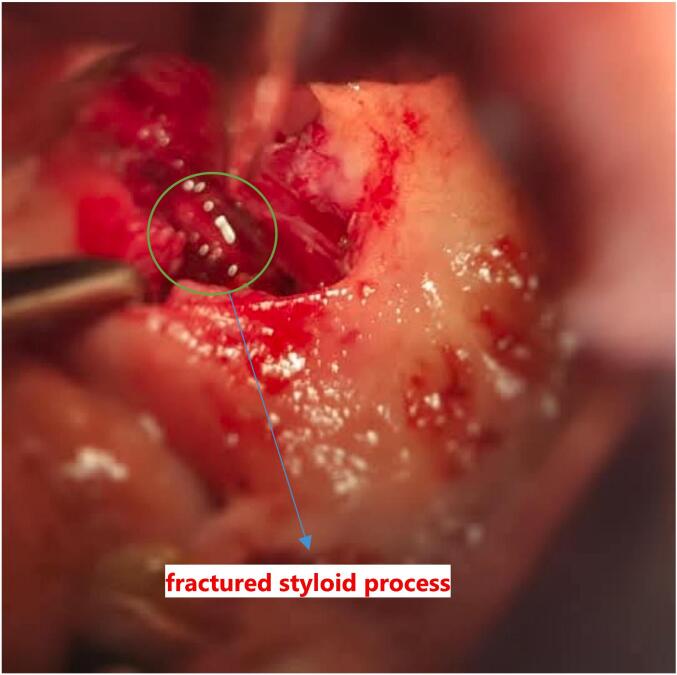
A clinical image after tonsillectomy and the dissecting proceeds across the superior constrictor muscle into the para-pharyngeal fascia space.

**Fig. 3:**
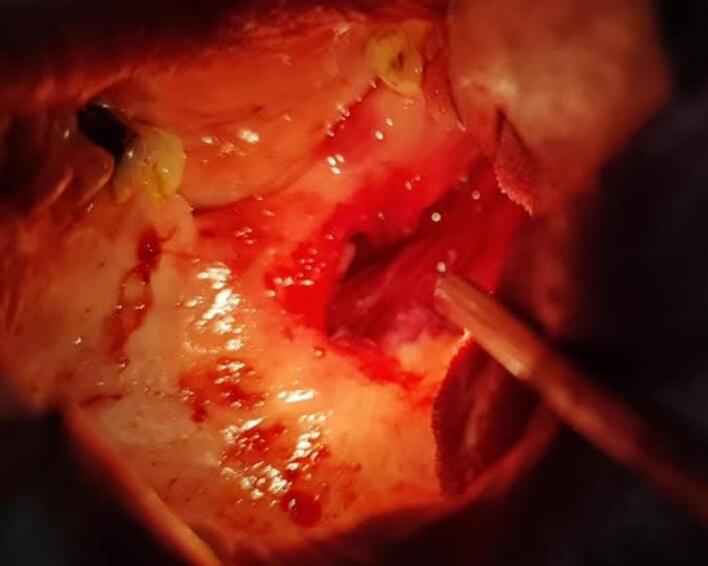
A clinical image demonstrates the location of styloid process after by-passing fat and para-pharyngeal fascia space.

**Fig. 4:**
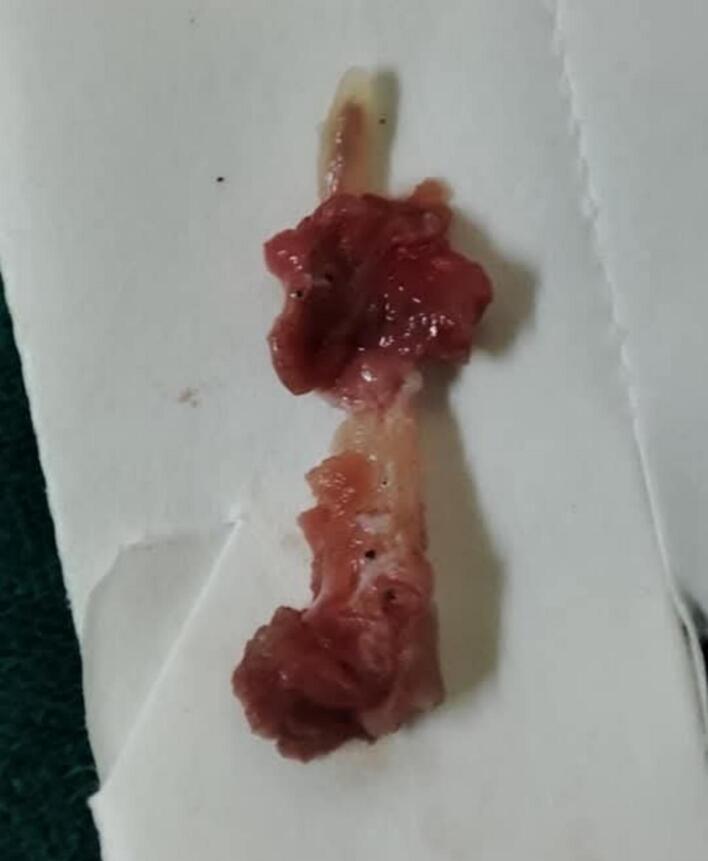
A clinical image displays the 46 mm elongated fractured styloid process with its calcified stylohyoid ligament.

The management of this case is done under general anesthesia, the Endoscopic-assisted Trans-oral approach is used for resection of the fractured stylohyoid process and calcified ligament. Initially, the tonsillectomy was performed first, then the superior constrictor muscle was perforated and divided (Fig. 2). After homeostasis, the fat is passed toward the anterior portion of the para-pharyngeal fascia. Here, the fractured styloid process was easily detected and located with finger palpation and endoscope assist (Fig. 3). Finally, the tonsillar bed was sutured after removal of fractured styloid process with calcified stylohyoid ligament (Fig. 4). The patient's symptoms were significantly improved after the surgery with no recurrence of pain in the subsequent 6 months follow - up period.

## 3 Discussion

Eagle's syndrome is associated with the presence of an abnormally elongated styloid process, potentially accompanied by an abnormal direction and/or ossification of the styloid ligament. The craniofacial pain experienced by patients resembles glossopharyngeal neuralgia and is attributed to irritation of surrounding neurovascular and muscular structures, including the carotid artery, cranial nerves, and muscles [1,2].

The condition often presents with mixed, non-specific symptoms, a lack of a clear etiology, and limited awareness of this syndrome, which can delay diagnosis. A thorough physical examination and clinical history are essential diagnostic tools. An elongated styloid process may be palpated during an intraoral examination and may elicit pain.

The differential diagnosis should consider conditions like glossopharyngeal and trigeminal neuralgia, temporal arteritis, migraine, cluster headache, myofascial pain dysfunction syndrome, pain from unerupted third molars, cervical arthritis and tumors [1].

Proper radiological evaluation, along with suitable technology and expertise, are critical to achieving an accurate diagnosis. CT scans of the head and neck, especially 3D-CT scans, are considered the gold standard for visualizing the anatomically complex styloid process. A diagnosis can be made not only using CT scan but also using plain radiography like panorama. There is some debate in the literature regarding how many patients with an elongated styloid process visible on radiologic examination do not exhibit clinical symptoms. Nevertheless, in confirmed cases of Eagle's syndrome [4,6].

The intraoral approach has advantages such as simplicity, shorter operation time, the feasibility of local anesthesia, and no visible external scarring. However, it has notable disadvantages, including limited access in cases of subsequent hemorrhage or deep neck infections, poor visualization of the surgical field (especially in patients with restricted jaw opening), the risk of iatrogenic injury to major neurovascular structures, and potential alterations in speech and swallowing due to postoperative swelling [7].

In contrast, the external approach offers better exposure of the styloid process and surrounding structures, which is a significant advantage. It also facilitates the removal of a partially ossified stylohyoid ligament. However, it tends to be more time-consuming, carries a risk of injury to the facial nerve and its branches, results in a visible neck scar, and may involve a longer recovery period. Ultimately, the choice of approach often depends on the surgical specialty of the operating surgeon. Surgical failure rates have been reported in up to 20 % of patients [7].

Warrier et all mentioned a case report of facial pain in a 22-year-old female patient who was diagnosed to have a unilateral elongated styloid process and the management was done using surgical excision of elongated styloid process [8].

Michaud et all reported an atypical case of bilateral elongated styloid process 75 mm in dental student and was management by bilateral styloidectomy [9].

In this case, we discussed a patient who did not respond to conservative management and was successfully treated using a surgical endoscopic-assisted trans-oral approach. While both trans-cervical and trans-oral methods are viable treatment options, we chose to use the endoscopic trans-oral approach removal, thereby improving visibility in the operating field, reducing complications, and achieving an ideal cosmetic and minimally invasive outcome.

## 4 Conclusion

Endoscopy-assisted Transoral resection is a minimally invasive surgical procedure for Eagle's syndrome that provides clear surgical view thus lessen the chance of neurovascular injury and other intraoperative complications.

## CRediT authorship contribution statement

FA: First surgeon and Supervision.

MJ: Assistant surgeon.

FI: Writing the manuscript and submission the manuscript.

JH: Writing the manuscript and submission the manuscript.

MA: Follow up.

All authors provided final approval of the version to be submitting.

## Consent

Written informed consent was obtained from the patient for publication and any accompanying images. A copy of the written consent is available for review by the Editor-in-Chief of this journal on request.

## Ethical approval

The ethical approval from our Scientific Research Board has been obtained.

## Ethics statement

The ethical approval from our institution Scientific Research Board Resolution- Tishreen University, Lattakia, Syria.

Board Status: Approved Approval number 1963–2024/12/21.

## Guarantor

Jafar Hamdy DDS, MSc, OMFS.

Firas Abdullrahman DDS, MSc, OMFS.

Authors Contribution:

FA: First surgeon and Supervision.

MJ: Assistant surgeon.

FI: writing the manuscript and submission the manuscript.

JH: writing the manuscript and submission the manuscript.

MA: follow up.

## Funding

No funding.

## Declaration of competing interest

There are no any financial and personal relationships with other people or Organizations.

## References

[1] Bokhari Maria R., Graham Charles, Mohseni Michael (Jan 2025).

[2] Saccomanno S., Greco F., DE Corso E., Lucidi D., Deli R., D’Addona A., Paludetti G. (Apr 2018). Eagle’s Syndrome, from clinical presentation to diagnosis and surgical treatment: a case report. Acta Otorhinolaryngol. Ital..

[3] Leming A.B., Vance D.G., Huang Z.J. (2024). Endoscopic-assisted treatment of pediatric Eagle Syndrome: a case report. Acta Oto-Laryngol. Case Rep..

[4] Badhey Arvind, Jategaonkar Ameya, Kovacs Alexander Joseph Anglin, Kadakia Sameep, De Deyn Peter Paul, Ducic Yadranko, Schantz Stimson, Shin Edward (2017). Eagle syndrome: a comprehensive review. Clin. Neurol. Neurosurg..

[5] Badheya Arvind, Jategaonkara Ameya, Kovacsb Alexander Joseph Anglin, Kadakiaa Sameep, De Deync Peter Paul, Ducicd Yadranko, Schantza Stimson, Shin Edward (May 2017). Eagle syndrome: a comprehensive review. Clin. Neurol. Neurosurg..

[6] Hassani M., Grønlund E.W., Albrechtsen S.S., Kondziella D. (2024). Neurological phenotypes and treatment outcomes in Eagle syndrome: systematic review and meta-analysis. PeerJ.

[7] Sohrabi C., Mathew G., Maria N., Kerwan A., Franchi T., Agha R.A. (2023). The SCARE 2023 guideline: updating consensus Surgical CAse REport (SCARE) guidelines. Int. J. Surg. Lond. Engl..

[8] Warrier S.A., Kc N., K S., Harini D.M. (Apr 10, 2019). Eagle’s syndrome: a case report of a unilateral elongated styloid process. Cureus.

[9] Michaud P.L., Gebril M. (Apr 26, 2021). A prolonged time to diagnosis due to misdiagnoses: a case report of an atypical presentation of Eagle syndrome. Am. J. Case Rep..

